# Anti-Invasive and Apoptotic Effect of Eupatilin on YD-10B Human Oral Squamous Carcinoma Cells

**DOI:** 10.3390/molecules30244666

**Published:** 2025-12-05

**Authors:** Gaeun Kim, Hyun-Jung Park, Suk-Yul Jung, Eun-Jung Kim

**Affiliations:** 1 Department of Biomedical Laboratory Science, College of Health Sciences, Sangji University, 83, Sangjidae-gil, Wonju 26339, Republic of Korea; 2 Department of Animal Biotechnology, College of Life Science, Sangji University, 83, Sangjidae-gil, Wonju 26339, Republic of Korea; 3 Department of Clinical and Diagnostic Sciences, Oakland University, 433 Meadow Brook Road, Rochester, MI 48309, USA

**Keywords:** eupatilin, oral squamous cell carcinoma, anti-invasion, apoptosis

## Abstract

Oral squamous cell carcinoma (OSCC) is an aggressive malignancy characterized by high invasiveness and poor prognosis. This study investigated the anticancer mechanisms of eupatilin, a pharmacologically active flavonoid derived from *Artemisia* species, in human OSCC YD-10B cells. Eupatilin significantly reduced cell viability in a dose-dependent manner, with an IC_50_ of approximately 50 μM. Flow cytometric analysis revealed G0/G1 phase arrest accompanied by downregulation of Cyclin D1 and CDK2, and upregulation of p21. Annexin V/Propidium Iodide staining and Western blotting confirmed apoptosis induction through activation of Bax, cleaved caspase-3/9, and poly ADP-ribose polymerase (PARP) cleavage, alongside suppression of Bcl-2. Furthermore, eupatilin markedly decreased both the mRNA expression and enzymatic activities of matrix metalloproteinases (MMP)-2 and MMP-9, indicating its potential to inhibit cancer cell invasion. Collectively, these findings demonstrate that eupatilin exerts potent antiproliferative and anti-invasive effects on OSCC cells via cell-cycle modulation and mitochondrial-mediated apoptosis. This study provides the first evidence of eupatilin’s therapeutic potential against OSCC, suggesting its promise as a natural compound for the development of safer and more effective treatments for oral cancer.

## 1. Introduction

Oral squamous cell carcinoma (OSCC) is a type of head and neck squamous cell carcinoma (HNSCC) and the most common neoplasm, accounting for approximately 90% of all oral cancers [1,2]. OSCC primarily arises in the oral cavity, including the tongue, gums, and oral mucosa, and is characterized by rapid proliferation, invasiveness, and a high recurrence rate. It is classified as a cancer with a poor prognosis due to a high risk of metastasis to the lungs, liver, and bones via lymph nodes. Early detection significantly improves survival rates, but delayed diagnosis complicates treatment and sharply reduces survival rates [3,4,5]. The global 5-year prevalence of OSCC in 2022 was 1,094,448, with an annual incidence and mortality rate of 389,846 and 188,438, respectively [6,7]. The incidence is particularly high in Asia, occurring more frequently in middle-aged and older men than in women [1,5].

OSCC is caused by the interaction of various environmental and genetic factors, such as smoking and drinking [8,9,10,11], betel quid intake [12,13], HPV infection [14,15], and chronic inflammation, and in some patients, it is preceded by oral potentially malignant disorders (OPMDs), such as leukoplakia, erythroplakia, oral submucous fibrosis, and oral lichen planus [16,17,18]. In this way, OSCC, which is caused by various causes, causes serious impairment in the patient’s basic life-sustaining functions such as breathing, chewing, swallowing, speaking, and taste, and significantly reduces the patient’s quality of life by accompanying changes in physical appearance during the tumor progression and treatment process [19]. In particular, tumor resection performed during the treatment of OSCC causes extensive tissue damage, resulting in various physical side effects, such as impaired swallowing, restricted tongue movement, oral pain, and decreased shoulder and arm function [20]. Furthermore, this functional damage goes beyond simple physical discomfort and can lead to communication difficulties and social withdrawal due to changes in appearance, which can lead to psychological distress and reduced social activities in patients.

Currently, standard treatments for OSCC include surgical resection, radiotherapy, and chemotherapy. However, these treatments have limitations, including low cure rates and serious side effects in advanced and recurrent cases. For early-stage OSCC (Stages I and II), surgery alone maintains a relatively high 5-year survival rate of 82%. However, for Stage III/IVA/IVB, the 5-year survival rate decreases to 52% even with surgery, high-dose radiotherapy, and chemotherapy, and if distant metastasis (Stage IVC) occurs, the 5-year survival rate is only 27%. Furthermore, 50% of patients with OSCC develop local recurrence after treatment, and in these cases, the median survival time is reported to be only 6–9 months [21,22].

The most commonly used chemotherapy for OSCC is cisplatin, typically combined with 5-FU (fluorouracil) as a platinum-based regimen. However, cisplatin-based chemotherapy can cause adverse effects such as nephrotoxicity, neuropathy, and myelosuppression, and can reduce overall patient compliance. Therefore, a new treatment strategy that can maximize tumor suppression effects while reducing the toxicity of existing anticancer drugs is needed, and natural plant-derived therapies are attracting attention as an alternative [23,24,25]. Natural substances currently reported for the treatment of OSCC include curcumin and chrysosplenol D. Curcumin, the main component of turmeric, has been reported to reduce tumor proliferation and invasion by inhibiting the EGFR signaling pathway in OSCC cells [26], and chrysosplenol D, derived from *Artemisia annua* L., has been shown to promote apoptosis by inhibiting the MAPK pathway and inducing heme oxygenase-1 expression in OSCC cells [27].

**Figure 1:**
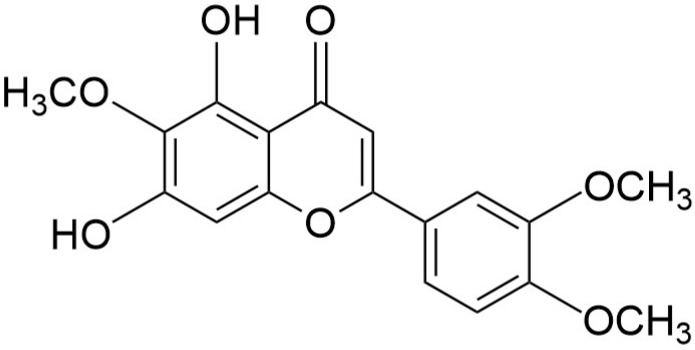
Chemical structure of eupatilin.

Flavonoids are a large group of plant-derived polyphenolic metabolites widely distributed in fruits, vegetables, and medicinal herbs, and they are well known for their antioxidant, anti-inflammatory, and anticancer properties [28]. Among these, hydroxy-methoxyflavones represent a subclass characterized by the presence of both hydroxyl and methoxy groups, structural features that enhance lipophilicity, metabolic stability, and overall biological efficacy [29]. Eupatilin (5,7-dihydroxy-3,4,6-trimethoxyflavone) is one of the representative hydroxy-methoxyflavones and is derived from plants of the genus *Artemisia*. Eupatilin, which contains multiple methoxy (-OCH_3_) and hydroxyl (-OH) groups, exhibits diverse physiological activities (Figure 1) and has been reported to have therapeutic potential in many diseases. First, eupatilin is known to modulate immune and inflammatory responses through its anti-inflammatory action. It reduces LPS-induced inflammatory cytokine expression by inhibiting the NF-κB and MAPK signaling pathways, and its effects have been confirmed in macrophage and bronchial epithelial cell models [30,31]. In addition, it has been reported to suppress liver and pulmonary fibrosis by regulating the β-catenin/PAI-1 and PI3K/Akt/mTOR signaling pathways through anti-fibrotic action [32,33]. Furthermore, it has an antioxidant function that protects cells from oxidative stress by suppressing the production of reactive oxygen species [34], and it has a particularly strong gastrointestinal protective effect, so it has been commercially used as a treatment for gastritis and gastric ulcers (Stillen™) [35]. These research results suggest that eupatilin is a natural substance with therapeutic potential in inflammatory, fibrotic, and metabolic diseases.

Recent studies have confirmed that eupatilin inhibits cancer cell proliferation and induces apoptosis, and thus, it is attracting attention as a candidate for anticancer drug development. In particular, it exhibited cytoprotective effects by inhibiting H_2_O_2_-induced apoptosis in gastric cancer and blocking ERK, JNK, and NF-κB signaling [36]. In esophageal cancer, it was reported to induce cell cycle arrest and suppress tumor growth by inhibiting Akt/GSK3β and MAPK/ERK signaling [37]. In addition, the mechanism of promoting cell death and increasing ROS production by inducing changes in mitochondrial membrane potential in melanoma was confirmed [38], and in renal cancer, it was shown to inhibit cancer cell proliferation and tumor growth by activating YAP1 signaling by suppressing the expression of mi-croRNA-21 [39].

Thus, eupatilin exhibits anticancer effects through modulation of signal transduction pathways in various cancer types, and is attracting attention as a promising natural compound that can suppress cancer cell proliferation while reducing the side effects of existing anticancer agents. Considering the need to develop new treatments for oral squamous cell carcinoma and the potential of eupatilin as an anticancer agent, this study aimed to demonstrate the anticancer effects of eupatilin in the YD-10B OSCC cell line.

## 2. Results

### 2.1. Effect of Eupatilin on the Viability of YD-10B Cells

**Figure 2:**
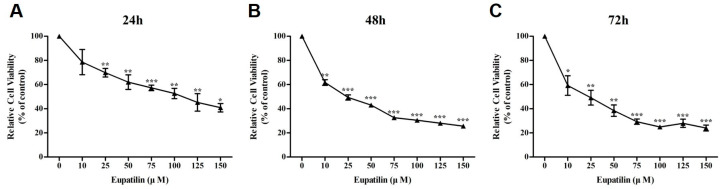
Anti-proliferation effect of eupatilin on YD-10B cells. The cells were treated with different concentrations of eupatilin for 24 h (**A**), 48 h (**B**) and 72 h (**C**). Data represent the mean ± S.D. through three independent experiments (*; *p* < 0.05 compared with untreated control, **; *p* < 0.01 compared with untreated control, ***; *p* < 0.001 compared with untreated control).

To evaluate the effect of eupatilin on the viability of the OSCC cell line YD-10B, an MTS assay was performed. Cells were treated with eupatilin at concentrations of 0, 10, 25, 50, 75, 100, 125, and 150 μM and cultured for 24, 48, and 72 h. The results showed a concentration-dependent decrease in cell viability in the eupatilin-treated group. The IC_50_ values were 68.79 μM at 24 h, 52.69 μM at 48 h, and 50.55 μM at 72 h, indicating a time-dependent increase in sensitivity to eupatilin (Figure 2). This confirmed that eupatilin effectively inhibits the survival of OSCC cell lines.

### 2.2. Cell Cycle Regulation by Eupatilin

**Figure 3:**
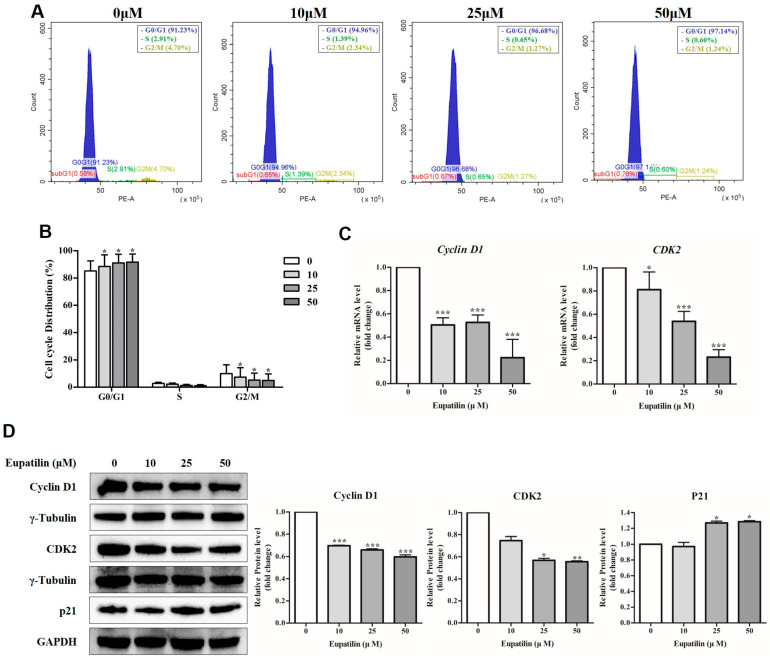
Cell cycle analysis of eupatilin on YD-10B cells. (**A**) Cell cycle distribution was analyzed by flow cytometry after treatment with various concentrations of eupatilin (0–50 μM) for 48 h. Histograms showed the proportion of cells in the G_0_/G_1_, S, and G_2_/M phases. The box in panel A indicates the cell cycle phase distribution (%). (**B**) Quantitative analysis was performed to determine the percentage of cells in each phase of the cell cycle after eupatilin treatment. (**C**) mRNA expression levels of *Cyclin D1* and *CDK2* were determined by RT-qPCR analysis. (**D**) Protein expression levels of Cyclin D1, CDK2, and p21 were examined by Western blot analysis. γ-Tubulin and GAPDH were used as loading controls. Relative band intensities were quantified using ImageJ (version 1.54g) software. Data represent the mean ± SD of three independent experiments (*; *p* < 0.05, **; *p* < 0.01, ***; *p* < 0.001 compared with untreated control).

To evaluate the effect of eupatilin on the cell cycle, YD-10B cells were treated with eupatilin at concentrations of 0, 10, 25, and 50 μM, cultured for 48 h, and the cell cycle distribution was analyzed using flow cytometry. The results showed that the proportion of cells arrested in the G0/G1 phase significantly increased in a concentration-dependent manner in the eupatilin-treated group, whereas the proportion of cells located in the S phase and G2/M phase significantly decreased as the eupatilin concentration increased (Figure 3A,B). These results suggest that eupatilin effectively inhibits cell division progression by inducing G0/G1 arrest. Analysis of the expression changes in Cyclin D1 and CDK2, key factors in regulating the G1/S transition, revealed that the mRNA and protein expression of *Cyclin D1* and *CDK2* decreased in a dose-dependent manner in the eupatilin-treated group (Figure 3C,D). In contrast, the protein expression of p21Waf1/Cip1, a cell cycle inhibitor, significantly increased (Figure 3D).

### 2.3. Induction of Apoptosis by Eupatilin

**Figure 4:**
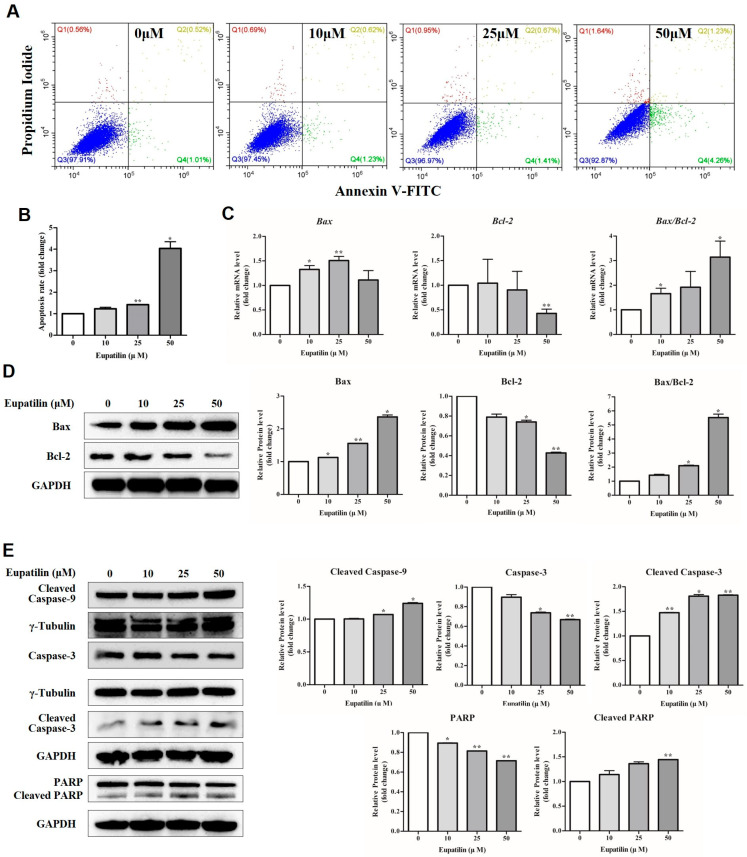
Effect of eupatilin on apoptosis in YD-10B cells. (**A**) Apoptotic cell death was analyzed using Annexin V-FITC/Propidium iodide (PI) double staining and flow cytometry after treatment with eupatilin (0–50 μM) for 48 h. The percentage of Annexin V-positive cells indicates apoptotic populations. (**B**) Quantification of apoptotic cells based on flow cytometric analysis. (**C**) mRNA expression levels of *Bax* and *Bcl-2* were determined by RT-qPCR, and the *Bax/Bcl-2* ratio was calculated. GAPDH was used as the loading control. (**D**) Protein expression levels of Bax and Bcl-2 were analyzed by Western blot analysis, and relative band intensities were quantified using ImageJ software. GAPDH was used as the loading control. (**E**) Expression of apoptosis-related proteins, including cleaved Caspase-9, Caspase-3, cleaved Caspase-3, PARP, and cleaved PARP, was evaluated by Western blot analysis. γ-tubulin and GAPDH were used as internal controls. Data represent the mean ± SD of three independent experiments (*; *p* < 0.05, **; *p* < 0.01 compared with untreated control).

To determine whether eupatilin induces apoptosis in YD-10B cells, Annexin V staining, RT-qPCR, and Western blot analysis were performed. After 48 h of treatment with eupatilin at concentrations of 0, 10, 25, and 50 μM, Annexin V staining was performed, and analysis revealed that Annexin V-positive cells increased approximately 4-fold compared to the control group at 50 μM (Figure 4A,B). This indicates early apoptotic cells, suggesting that eupatilin induces cell death through apoptosis in YD-10B cells. To analyze changes in the expression of apoptosis-related genes and proteins, RT-qPCR and Western blot were performed. As a result, the expression of Bax, a pro-apoptotic factor, increased in the eupatilin-treated group, while the expression of Bcl-2, an anti-apoptotic factor, significantly decreased. Accordingly, the Bax/Bcl-2 ratio showed a concentration-dependent increase, with the highest ratio observed at 50 μM (Figure 4C,D). Western blot analysis was performed to check the activation of the caspase family, a key protein in the mitochondrial (intrinsic) apoptosis pathway. As a result, as the concentration of eupatilin increased, the cleaved forms of caspase-9, an initiator caspase, and caspase-3, an executioner caspase, increased, while the pro-form decreased. In addition, poly ADP-ribose polymerase (PARP), a major substrate of caspase-3, also showed an increased expression of the cleaved form and a promoted activity pattern (Figure 4E).

### 2.4. Effect of Eupatilin on Invasion of YD-10B Cells

**Figure 5:**
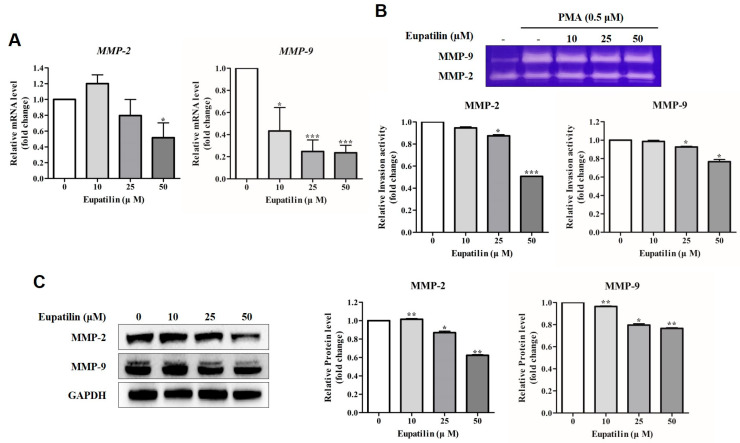
Effect of eupatilin on the expression of MMP-2/9 in YD-10B cells. (**A**) Effect of eupatilin on the mRNA expression level of MMP-2/9 mRNA in PMA-treated YD-10B cells. (**B**) The activity of MMP-2/-9 protein was determined by gelatin zymography in conditioned media. (**C**) Protein expression levels of MMP-2 and MMP-9 were determined by Western blot analysis. GAPDH was used as a loading control. The relative expression of MMP-2/-9 was analyzed by band intensity using ImageJ program. Data represent similar results from three independent experiments (*; *p* < 0.05, **; *p* < 0.01, ***; *p* < 0.001 compared with untreated control).

In this study, we evaluated the inhibitory effect of eupatilin on matrix metalloproteinases (MMP) expression induced by the tumor promoter phorbol 12-myristate 13-acetate (PMA). YD-10B cells were treated with PMA (0.5 μM) alone or in combination with eupatilin (10, 25, or 50 μM) for 24 h, and then RT-qPCR and gelatin zymography were performed. RT-qPCR results showed that the mRNA expression of MMP-2 and MMP-9 decreased in a concentration-dependent manner in the eupatilin-treated group (Figure 5A), and gelatin zymography also showed that the gelatinolytic activity of MMP-2 and MMP-9 was significantly inhibited as the eupatilin concentration increased (Figure 5B). Western blot results also showed that the protein expression of MMP-2 and MMP-9 tended to decrease depending on the eupatilin treatment concentration (Figure 5C).

## 3. Discussion

In this study, the biological effects of eupatilin, a natural flavonoid, on the YD-10B cell line were elucidated. Eupatilin exhibited anticancer activity through inhibition of cell proliferation, cell cycle regulation, induction of apoptosis, and inhibition of cell invasion. Eupatilin decreased the viability of YD-10B cells in a concentration-dependent manner, with an IC_50_ value of 68.79 μM at 24 h, 52.69 μM at 48 h, and 50.55 μM at 72 h. These results were consistent with the anticancer effects of eupatilin reported in other cancer types. Fei et al. observed a significant decrease in cell viability in a concentration- and time-dependent manner when treating glioma cell lines (U251MG, U87MG, T98G, U118) with eupatilin at concentrations of 40–320 μM [40], which showed a similar trend to the pattern observed in YD-10B cells in this study. Furthermore, Zhong et al. reported that treatment with eupatilin at 20 μM and 40 μM in renal carcinoma cells (786-O) resulted in a reduction in cell viability by 47.2% and 61.3%, respectively [41]. These previous findings suggest that eupatilin exerts broad inhibitory effects on cell proliferation across various cancer types, thereby supporting the validity of the anticancer effects against OSCC demonstrated in the present study.

Flow cytometry analysis showed that the proportion of cells in the G0/G1 phase increased and the proportions of cells in the S phase and G2/M phase decreased in the eupatilin-treated group, confirming the induction of G0/G1 arrest. At the molecular level, eupatilin decreased the expression of Cyclin D1 and CDK2, which are involved in cell cycle progression, and increased the expression of p21, a cell cycle inhibitor. Cyclin D1 is generally known to form a complex with CDK4/6, and Cyclin E is known to bind to CDK2 to regulate the cell cycle. However, previous studies have shown that Cyclin D1 can also form a complex with CDK2. This Cyclin D1-CDK2 complex phosphorylates at Thr160, the CDK activation site, leading to multi-site phosphorylation of the Rb protein. Furthermore, the complex mediates E2F-dependent transcriptional activation, enabling recognition of both CDK4 and CDK2 substrates, demonstrating dual functional properties associated with cell proliferation and tumor cell transformation [42]. In addition, it has been reported that the Cyclin D family can regulate CDK2 activity in addition to CDK4/6 [43] and that p21, a CIP/KIP family inhibitor, redistributes between the Cyclin D–CDK4/6 complex and the Cyclin E–CDK2 axis to suppress CDK2 activity, thereby regulating the G1/S transition [44]. These results suggest that Cyclin D1 and CDK2 do not act independently but rather interact in a complex manner to precisely regulate cell cycle progression. Therefore, eupatilin may inhibit the formation or activity of the Cyclin D1–CDK2 complex while simultaneously inducing p21Waf1/Cip1 expression, thereby blocking G1/S transition and contributing to eupatilin-induced G0/G1 arrest.

When evaluating apoptosis induction by eupatilin, the number of Annexin V-positive cells increased approximately 4-fold in the 50 μM eupatilin-treated group compared to the control group. This suggests that phosphatidylserine, translocated to the outer side of the cell membrane during the early stage of apoptosis, binds to Annexin V. Analysis of the expression of apoptosis-related factors revealed that the pro-apoptotic factor Bax increased, while the anti-apoptotic factor Bcl-2 decreased, resulting in a significant increase in the Bax/Bcl-2 ratio. Furthermore, the expression of cleaved caspase-9, cleaved caspase-3, and cleaved PARP increased, demonstrating that eupatilin induced apoptosis through the mitochondrial pathway. Notably, the mitochondrial apoptosis-inducing effect of eupatilin confirmed in this study was consistent with the results of previous studies reported in other cancers. Lee et al. demonstrated that eupatilin activates the mitochondrial apoptotic pathway in osteosarcoma cell lines (U-2 OS) through an increase in the Bax/Bcl-2 ratio, a decrease in mitochondrial membrane potential, cytochrome c release, and caspase-3/9 activation and PARP cleavage [45]. Seo et al. reported that treatment with eupatilin in human promyelocytic leukemia HL-60 cells resulted in the release of cytochrome c from mitochondria into the cytoplasm, followed by the sequential activation of caspase-9, -3, and -7 and PARP cleavage, leading to the progression of apoptosis [46]. Taken together, these results indicate that the observed mechanism of eupatilin-induced apoptosis in YD-10B cells in this study was consistent with these previous studies, suggesting that eupatilin is an anticancer agent that activates the mitochondria-dependent apoptotic pathway in various cancers. Although activation of Bax/Bcl-2 signaling, caspase-9/3 cleavage, and PARP fragmentation clearly demonstrates the involvement of mitochondria-mediated apoptosis, the proportion of Annexin V–positive cells observed in this study remained relatively low (5.5% at 50 μM). This suggests that apoptosis alone may not fully account for the eupatilin-induced cytotoxicity in YD-10B cells. Previous reports have indicated that eupatilin can trigger additional modes of regulated cell death, including mitochondrial dysfunction–associated ROS accumulation, autophagy-related signaling alterations, and non-apoptotic cytotoxic mechanisms in other cancer models. Therefore, it is plausible that eupatilin may simultaneously activate alternative cell death pathways—such as autophagy-dependent cell death, necroptosis, or ferroptosis—alongside apoptosis in OSCC cells. Further studies involving pathway-specific inhibitors and molecular markers will be required to clarify the relative contribution of each cell death modality to the overall anticancer effect of eupatilin.

To evaluate the inhibition of cell invasion by eupatilin, RT-qPCR, gelatin zymography, and Western blot analyses were performed. Eupatilin dose-dependently reduced the mRNA and protein expression of MMP-2 and MMP-9, and also inhibited their gelatinolytic activity. MMPs are zinc-dependent endogenous proteases that are involved in tumor cell invasion and metastasis through extracellular matrix degradation [47,48]. In addition to extracellular matrix degradation, MMPs are also involved in various physiological processes such as cell adhesion, signal transduction, and immune regulation, and are known to contribute to the initial growth and invasion of cancer cells by degrading proteins that constitute the basement membrane [49]. MMPs are classified into collagenases (MMP-1, -8, -13, -18), gelatinases (MMP-2, -9), stromelysins (MMP-3, -10, -11), and matrilysins (MMP-7, -26) based on their structural characteristics and substrate preferences [50,51]. Among these, MMP-2 and MMP-9 are key proteins that promote cancer cell invasion by degrading type IV collagen, a major component of the basement membrane. It was confirmed that eupatilin effectively inhibits cell invasion by suppressing both the expression and activity of MMP-2 and MMP-9. The MMP inhibitory effect of eupatilin has also been reported in other cancer types. Jeong et al. reported that eupatilin inhibited cell invasion in the gastric cancer MKN-1 cell line by reducing the expression of MMP-2 and MMP-9 and gelatinolytic activity through inhibition of NF-κB activity [52]. In addition, Fei et al. showed that eupatilin inhibits cell invasion by regulating the RECK/MMP pathway in glioblastoma, and the inhibitory effect on MMP-2 was significant, but relatively weak on MMP-9, suggesting the possibility of a differential regulatory mechanism for each cancer type [40]. These previous studies suggest that eupatilin has a common anticancer mechanism of blocking invasion and metastasis through inhibition of MMP activity in various cancers, which was consistent with the MMP inhibitory effect observed in YD-10B cells in this study.

The importance of apoptosis induction in cancer treatment has been demonstrated in numerous studies. Cancer cells typically proliferate continuously through cell cycle dysregulation and activation of anti-apoptotic signaling pathways, often resulting in resistance to conventional chemotherapeutic agents and radiation therapy [53,54]. Therefore, strategies that induce apoptosis are considered important therapeutic approaches because apoptosis proceeds in a non-inflammatory manner, thereby minimizing damage to surrounding normal cells. The finding in this study that eupatilin inhibited OSCC cell survival by activating the intrinsic apoptotic pathway suggests that eupatilin may be a useful candidate for the treatment of OSCC.

Currently, most cancer treatments, including OSCC, use various chemotherapeutic agents that target mechanisms such as inhibition of cell proliferation, induction of apoptosis, and inhibition of invasion and metastasis. However, drug resistance and serious side effects are pointed out as major limitations. In recent studies, natural products have been attracting attention as complementary treatments because they have similar effects to existing treatment mechanisms, while also lowering the possibility of developing resistance and reducing side effects [26,27,55]. Because existing chemotherapeutic agents work by blocking a specific single target, cancer cells are more likely to acquire resistance by activating alternative signaling pathways, and they can also affect normal cells, causing side effects such as immune suppression, liver toxicity, and kidney damage. In contrast, natural compounds possess low toxicity and exhibit relatively low toxicity to normal cells, ensuring stability even in long-term treatment [53,56]. Furthermore, they are attracting attention as a combination therapy with existing chemotherapeutic agents, as natural products can enhance the therapeutic efficacy of existing anticancer agents while reducing side effects. Studies have reported maximizing therapeutic efficacy in OSCC through combination therapy with cisplatin, a representative chemotherapeutic agent, and various natural products [57,58]. This suggests that natural product-based treatments can provide a more effective treatment strategy, not only as monotherapy but also in combination with existing treatments.

## 4. Materials and Methods

### 4.1. Materials

YD-10B OSCC cells were purchased from the Korea Cell Line Bank (Seoul, Republic of Korea). RPMI-1640, fetal bovine serum (FBS), trypsin-EDTA solution, and penicillin/streptomycin solution were purchased from Gibco-BRL (Grand Island, NY, USA). Eupatilin was purchased from Sigma-Aldrich (St. Louis, MO, USA).

### 4.2. Cell Culture and Eupatilin Treatment

YD-10B cells were cultured in RPMI-1640 containing 10% fetal bovine serum and 1% antibiotics (100 U/mL for penicillin, 100 μg/mL for streptomycin) in an incubator at 37 °C and 5% CO_2_. Eupatilin was dissolved in dimethyl sulfoxide to prepare a 100 mM stock solution and stored at −80 °C. For cell treatment, the stock solution was diluted in RPMI-1640 to the indicated concentrations, ensuring that the final concentration of DMSO in all treatment groups was less than 0.1%.

### 4.3. Dimethylthiazole-2′, 5′-Diphenyl-2-H-Tetrazlium Bromide (MTS) Assay

To investigate cell viability, it was measured using the EZ-3000 assay kit (DoGENBIO, Seoul, Republic of Korea). YD-10B cells were seeded at 2 × 10^4^ cells per well in a 96-well plate and cultured for 12 h. Then, eupatilin was treated at concentrations of 10, 25, 50, 75, 100, 125, and 150 μM. The cells were cultured in an incubator at 37 °C and 5% CO_2_ for 24, 48, and 72 h. After that, 10 μL/well of MTS solution was added to the cell culture medium and reacted in an incubator at 37 °C for 1 h. The absorbance was measured at 450 nm using a microplate reader (Molecular Devices, Sunnydale, CA, USA).

### 4.4. Cell Cycle Analysis

YD-10B cells were seeded in 60 mm culture dishes at 1 × 10^6^ cells/well and cultured for 18 h. Then, eupatilin was treated at concentrations of 10, 25, and 50 μM and cultured for 48 h. The cells were trypsinized, washed with phosphate-buffered saline (PBS), and fixed with 70% ethanol at 4 °C for 12 h. The fixed cells were washed with PBS and treated with 100 μg/mL RNase A at 37 °C for 30 min. The cells were stained with 50 μg/mL Propidium Iodide, and the cell cycle was monitored using a CytoFLEX Flow Cytometer (Beckman Coulter Life Sciences, Brea, CA, USA).

### 4.5. Annexin V-FITC Analysis

YD-10B cells were seeded at 1 × 10^6^ cells/well in 60 mm culture dishes and cultured for 12 h. Then, the cells were treated with various concentrations of eupatilin and cultured for 48 h. The cells were collected, washed with PBS, stained using the Annexin V-FITC Apoptosis kit (Biovision Inc., Milpitas, CA, USA), and measured using a CytoFLEX Flow Cytometer (Beckman Coulter Life Sciences, CA, USA).

### 4.6. Reverse Transcription Quantitative Polymerase Chain Reaction (RT-qPCR)

Total RNA was extracted from cells using TRIzol reagent (Invitrogen Co., Carlsbad, CA, USA). Reverse transcription of 1 μg of RNA was performed using the ReverTra ACE PCR RT Master Mix Kit (TOYOBO Co., Osaka, Japan) to synthesize cDNA. For quantitative gene expression analysis, primers for each gene were used at a concentration of 1 pmole, and the reaction solution was prepared with SYBR Green (Applied Biosystems, Foster City, CA, USA) reagent to a total reaction volume of 10 μL. PCR reactions were performed using the QuantStudio™ 3 Real-Time PCR System (Applied Biosystems). The relative mRNA expression levels of cyclin D1, CDK2, MMP-2, and MMP-9 were calculated using the 2^−ΔΔCt^ method and normalized to GAPDH expression. The primer sequences for each gene are as follows: MMP-2 (forward: 5′-ccaccacctacaactttgaga-3′, reverse: 5′-cgacaagaagtatggcttctg-3′), MMP-9 (forward: 5′-acaagctcttcggcttctg-3′, reverse: 5′-gaactttgacagcgacaagaa-3′), Cyclin D1 (forward: 5′-gctgcgaagtggaaaccatc-3′, reverse: 5′-ttcaaatgtgtgcagaaggagg-3′), CDK2 (forward: 5′-aacacagagggggccatcaagc-3′, reverse: 5′-gaccctgtggtaccgagctcctg-3′), GAPDH, which was used as an internal control (forward: 5′-gaaggtgaaggtcggagt-3′, reverse: 5′-gaagatggtgatgggatttc-3′).

### 4.7. Western Blot

YD-10B cells treated with eupatilin at various concentrations were collected, washed with PBS, and lysis buffer (150 mM sodium chloride, 1.0% NP-40, 0.5% sodium deoxycholate, 0.1% sodium dodecyl sulfate, 50 mM Tris-Cl, pH 8.0) was added. The cells were incubated at 4 °C for 15 min, centrifuged at 12,000 rpm for 10 min, and the supernatant was collected. Proteins were quantified using the Bradford method, and 50 μg of protein were separated using SDS-PAGE gel. After that, the cells were transferred to a polyvinylidene difluoride membrane and incubated at room temperature for 1 h in TBS-T buffer (20 mM Tris-Cl, pH 7.4, 150 mM NaCl, 0.1% Tween-20) containing 5% skim milk. Cyclin D1 (#A19038; Abclonal, Wuhan, China), CDK2 (#2546; Cell Signaling Technology, Danvers, MA, USA), p21 (#2947; Cell Signaling Technology), Bax (#14796, Cell Signaling Technology), Bcl-2 (#15071; Cell Signaling Technology), caspase-9 (#A18676; Abclonal) (#9542; Cell Signaling Technology), caspase-3 (#9662; Cell Signaling Technology), cleaved caspase-3 (#9661; Cell Signaling Technology), PARP (#9542; Cell Signaling Technology), MMP-2 (#A6247; Abclonal), MMP-9 (#A0289, Abclonal), γ-Tubulin (#sc-7396; Santa Cruz Biotechnology, Santa Cruz, CA, USA), and GAPDH (#AC033; Abclonal) were diluted 1:1000 and added and reacted at 4 °C for 12 h. Goat anti-mouse horseradish peroxidase (HRP)-conjugated IgG secondary antibody (#7074P2; Cell Signaling Technology), Horse anti-mouse HRP-conjugated IgG secondary antibody (#7076P2; Cell Signaling Technology), and Donkey anti-goat HRP-conjugated IgG secondary antibody (#sc-2020; Santa Cruz Biotechnology) were diluted 1:5000 and reacted at room temperature for 1 h. After that, the chemiluminescence was measured using an enhanced chemiluminescence (ECL) kit (Amersham pharmacia Biotech Ltd., Amersham, UK).

### 4.8. Gelatin Zymography

YD-10B (1 × 10^6^) cells were cultured for 24 h, treated with various concentrations of eupatilin and the tumor promoter phorbol 12-myristate 13-acetate (PMA) at 0.5 μM, either alone or in combination. The supernatant was collected and concentrated using an Ultra-4-Centrifugal Filter Unit (Merck Millipore, Temecula, CA, USA). 20 μg of protein was mixed with non-reducing sample buffer (0.5 M Tris-Cl, pH 6.8, 5% SDS, 20% glycerol, 1% bromophenol blue) and subjected to electrophoresis on a 10% sodium dodecyl sulfate-polyacrylamide gel electrophoresis (SDS-PAGE) gel containing 1% gelatin. After electrophoresis, SDS was removed with washing buffer (2.5% Triton X-100), and the gel was incubated with incubation buffer (1 M Tris-Cl pH 7.5, 1 M CaCl_2_, 5 M NaCl, 0.02% NaN_3_, 0.2 mM ZnCl_2_, 2.5% Triton X-100) at 37 °C for 12 h. The gel was stained with Coomassie brilliant blue (7% glacial acetic acid, 40% methanol, 0.25% Coomassie blue) for 30 min, and then destained with destaining solution (7% glacial acetic acid, 40% methanol) to confirm the white band.

### 4.9. Statistical Analysis

All experiments were repeated three times and expressed as mean ± SD. Student’s *t*-test was performed to obtain statistically significant results between each experimental group.

## 5. Conclusions

This study evaluated the anticancer effects of eupatilin in vitro and is of academic significance in that it is the first to report its apoptosis-inducing and invasion-inhibiting effects in YD-10B OSCC. Eupatilin dose-dependently decreased the viability of YD-10B cells and induced G0/G1 cell arrest through decreased expression of cyclin D1 and CDK2 and increased expression of p21. Furthermore, it activated the mitochondrial-mediated apoptosis pathway, promoting an increase in the Bax/Bcl-2 ratio and caspase-9/3 and PARP cleavage. It also inhibited the expression and activity of MMP-2 and MMP-9, thereby blocking the invasive ability of cancer cells. These results demonstrate that eupatilin effectively inhibits OSCC through a multifaceted anticancer mechanism of cell cycle regulation, apoptosis induction, and invasion inhibition. Considering the serious side effects and resistance development of existing chemotherapy, eupatilin, with its low toxicity, could be a safer alternative treatment strategy. However, this study was limited to an in vitro model. For clinical application, further research is needed, including in vivo efficacy verification, bioavailability and pharmacokinetic analysis, and evaluation of the efficacy of combination therapy with existing anticancer agents. In conclusion, this study suggests that eupatilin is a promising candidate for a natural anticancer agent for the treatment of OSCC, and provides a basis for the development of a new therapeutic strategy that may contribute to improving the treatment outcomes and quality of life of OSCC patients in the future.

## Data Availability

The original contributions presented in this study are included in the article. Further inquiries can be directed to the corresponding authors.
